# The Impact of the Online COVID-19 Infodemic on French Red Cross Actors’ Field Engagement and Protective Behaviors: Mixed Methods Study

**DOI:** 10.2196/27472

**Published:** 2021-10-06

**Authors:** Leonardo W Heyerdahl, Benedetta Lana, Tamara Giles-Vernick

**Affiliations:** 1 Department of Global Health, Anthropology and Ecology of Disease Emergence Unit Institut Pasteur Paris France

**Keywords:** COVID-19, infodemics, social listening, epidemics, medical anthropology, nongovernmental organizations

## Abstract

**Background:**

The COVID-19 pandemic has been widely described as an infodemic, an excess of rapidly circulating information in social and traditional media in which some information may be erroneous, contradictory, or inaccurate. One key theme cutting across many infodemic analyses is that it stymies users’ capacities to identify appropriate information and guidelines, encourages them to take inappropriate or even harmful actions, and should be managed through multiple transdisciplinary approaches. Yet, investigations demonstrating how the COVID-19 information ecosystem influences complex public decision making and behavior offline are relatively few.

**Objective:**

The aim of this study was to investigate whether information reported through the social media channel Twitter, linked articles and websites, and selected traditional media affected the risk perception, engagement in field activities, and protective behaviors of French Red Cross (FRC) volunteers and health workers in the Paris region of France from June to October 2020.

**Methods:**

We used a hybrid approach that blended online and offline data. We tracked daily Twitter discussions and selected traditional media in France for 7 months, qualitatively evaluating COVID-19 claims and debates about nonpharmaceutical protective measures. We conducted 24 semistructured interviews with FRC workers and volunteers.

**Results:**

Social and traditional media debates about viral risks and nonpharmaceutical interventions fanned anxieties among FRC volunteers and workers. Decisions to continue conducting FRC field activities and daily protective practices were also influenced by other factors unrelated to the infodemic: familial and social obligations, gender expectations, financial pressures, FRC rules and communications, state regulations, and relationships with coworkers. Some respondents developed strategies for “tuning out” social and traditional media.

**Conclusions:**

This study suggests that during the COVID-19 pandemic, the information ecosystem may be just one among multiple influences on one group’s offline perceptions and behavior. Measures to address users who have disengaged from online sources of health information and who rely on social relationships to obtain information are needed. Tuning out can potentially lead to less informed decision making, leading to worse health outcomes.

## Introduction

One critical concern emerging during the COVID-19 pandemic has been the infodemic, defined as excessive information that spreads rapidly, may be deliberately or inadvertently misleading, complicates emergency risk communication, and encourages lay publics to engage in harmful actions during this public health emergency [1-5]. Neither this phenomenon during epidemics nor investigations of it are new. Infodemiology—“the study of the determinants and distribution of health information and misinformation”—emerged in the late 20th century and was shortly thereafter conceptualized as a field of study [6,7]. Infodemics as informational companions to epidemics developed with the first SARS epidemic and continued subsequently during the H1N1, Ebola, and Zika public health emergencies [8-10]. The World Health Organization Director General popularized the term in the context of the COVID-19 pandemic, declaring, “We are not just fighting an epidemic; we’re fighting an infodemic” [11].

The current pandemic has catalyzed numerous social media–listening investigations to monitor, evaluate, and respond to circulating misinformation and disinformation during this infodemic [4,12-14]. In identifying problematic narratives and measuring their online spread, one key theme cutting across many such analyses is that the infodemic is a threat; it rapidly overwhelms users with contradictory and misleading information and encourages them to make risky or harmful decisions [5,6,15-19].

How this complex information ecosystem influences offline behavior—real-life choices and practices—is a critically important question during the COVID-19 pandemic, although it remains insufficiently investigated [20]. Media analyses, psychology, and anthropology have addressed this interaction between online information interpretation and offline behavior differently.

Drawing from the fields of communication, marketing, and computer science, social media analyses have detected the emergence and spread of COVID-19 misinformation and characterized narratives and concerns of users [21-24]. Such analyses can shed important light on public concerns about public health measures, yet they offer less insight into real-life practice [25,26]. Islam and colleagues found a correlation between online stigmatization of, and offline violence toward, Asian populations [27]. Although such correlations are compelling, they do not question the specific agents of this violence with the aim to understand how they obtained and interpreted online narratives and decided to act upon them.

Behavioral studies drawing from psychology often measure the influence of social and traditional media on psychological states or on health behaviors [28-34]. During the COVID-19 pandemic, these studies focused primarily on misinformation and disinformation, evaluating the language of media users or employing closed questionnaires to assess subjects’ media use and offline emotions and behaviors [35-43]. For these researchers, attending to cultural differences (eg, around mask use) can illuminate divergent psychological states [44]. These studies yield important insights into the psychological impact of misinformation and disinformation. In concentrating on misinformation and disinformation, they characterize and aggregate individual responses, but risk neglecting underlying sociocultural, political, and economic conditions that may inform the emotional and behavioral responses of specific social groups.

Although not well known by other fields, anthropological contributions to evaluating rumors and infodemics are two-fold. First, over the past three decades, anthropologists and ethnohistorians addressing epidemic crises and biomedical research have been less preoccupied with distinguishing truth from rumors than with understanding specific social groups’ perceptions and actions, and the factors shaping them [45-50]. Tappan [46] and Graboyes [47], in particular, contend that rumors of blood theft offer rich insight into East Africans’ criticisms of biomedical research, in addition to reflections on the political, economic, and social inequalities that late colonialism imposed. This anthropological perspective on rumors, and more broadly an insistence on the valuable insights drawn from evaluating all information, rather than sifting out misinformation and disinformation from a broader body of circulating information, has informed both online ethnographies and research on the current COVID-19 infodemic [8,51-54]. A second contribution of anthropology, as well as other field-based qualitative social sciences, is a preoccupation with situating a social group’s specific understandings and practices within their broader sociocultural, political, and economic contexts [55]. This preoccupation necessitates the use of multiple methods that permit anthropologists to capture what informants say, think, and do, and to gain insight into the multiple influences that shape those words, thoughts, and actions.

These two anthropological concerns frame our central question and our approach in this study. Our question focuses on how the COVID-19 infodemic has affected offline public behavior. We investigated whether and how social and traditional media influenced risk perceptions and behaviors of a heterogeneous social group in the Paris region of France: French Red Cross (FRC) volunteers and workers. Specifically, we conducted quantitative and qualitative social listening and analysis of Twitter, its links to other media, and selected print media in France from April to October 2020. This popular microblogging site, its links, and selected print media served as a proxy for public debates around COVID-19. Simultaneously, we evaluated how FRC volunteers and workers experienced and portrayed the influence of social and traditional media on their risk perceptions, their engagement in FRC field actions, and their protective behaviors.

## Methods

### Site and Population Description

This study has been carried out in the Ile-de-France region (Paris region) of France, a region comprised of eight departments that cover the city of Paris and its suburbs, with a population of 12.1 million people.

The population investigated in the study consisted of FRC salaried workers and volunteers carrying out operations in the Ile-de-France region. The FRC is a nongovernmental organization providing critical support for France’s public health system during the COVID-19 pandemic, organizing and conducting diagnostic testing and emergency medical services. An estimated 42,800 volunteers in this region are mostly lay people undertaking social assistance activities (eg, food assistance; outreach for elderly, homeless, or other vulnerable populations; and support for school-age children), but a small proportion (less than 5%) serve as rescue workers, physicians, nurses, nursing assistants, and technicians performing emergency and first aid response and providing health care. The FRC’s 4200 salaried workers provide medical, material, and legal assistance to vulnerable populations in specialized centers and manage the logistics of FRC activities, as well as financial and other donations. The FRC’s major presence in the humanitarian landscape makes it a useful organization to investigate during the pandemic.

The FRC was also selected because in early March 2020, prior to France’s first lockdown, the French Red Cross Foundation contacted our research team, requesting that we investigate how the COVID-19 pandemic affected FRC workers and volunteers. Given that the COVID-19 infodemic was a serious global concern, we eventually decided to explore whether the changing information ecosystem affected workers’ and volunteers’ motivations: their decisions to participate in FRC field activities, as well as their self-protective measures.

### Data Collection

#### Twitter Social Listening and Selected Media Evaluation

We collected data through Twitter and selected traditional media to capture a range of COVID-19 public health debates. Social and traditional media form part of an informational ecosystem: social and traditional media mutually influence one another and, at times, overlap [56].

From April to October 2020, we conducted social listening of COVID-19–related messages on Twitter and tracked COVID-19–related debates in traditional media. Using custom scripts in R (version 4.0.2; The R Foundation) and the rtweet package, we submitted daily keyword-based “coronavirus OR COVID” queries to the Twitter application programming interface. All matching organic tweets—not retweets—in French from accounts declaring a France-based location were collected.

Additional queries were added based on emerging themes from interviews or from traditional print media. These queries addressed lockdowns (keyword: “confinement”), hydroxychloroquine (“hydroxychloroquine”), vaccines (“vaccin”), and masks (“masque”). Such queries characterized narratives about epidemic events and identified scientific and public health debates emerging from interviews or from Twitter data.

To supplement this source, we also viewed linked sources (eg, articles, videos, radio programs, and websites) and followed three of the top five daily newspapers covering a range of political positions—*Le Monde*, *Libération,* and *Le Figaro—*to identify changing debates around COVID-19 [57]. Linked sources provided additional contextual information about the tweet and provided additional content on new debates. We also participated in an international WhatsApp group of researchers sharing diverse media sources from around the world to track new and ongoing pandemic debates. These combined sources served as a proxy for key public debates over public health measures and biomedical investigations; they supplemented our inquiries in Twitter and contributed specific questions about online and media debates concerning the pandemic in our qualitative interviews.

#### Semistructured Interviews

From June to October 2020, we conducted 24 semistructured individual interviews with FRC volunteers and workers. The interview guide (Multimedia Appendix 1) addresses informants’ training, their activities before the pandemic, and how these activities changed with pandemic emergence, lockdown measures, and deconfinement. Crucially, it explored participants’ risk perceptions of COVID-19 and their decisions to participate or not in FRC activities. The interview guide also included questions about traditional and social media and the informant’s use of them.

Our analyses of social listening and media tracking enabled us to ask specific interview questions about whether and how specific traditional and social media debates had influenced a participant’s emotional responses, decisions, or practices. Even if individual participants did not use Twitter specifically, we nevertheless asked questions about their understanding and interpretation of current public debates over public health measures and biomedical investigations, as well as whether these debates influenced their risk assessments, their decisions to continue field FRC activities, and their adherence to protective measures. All interviews were conducted in French and recorded with informant consent.

### Recruitment

The FRC compiled a randomly selected database of 9000 volunteers and workers in the Ile-de-France region, stratified by proportion of volunteers and workers, gender, department, and age. We recruited interview participants by randomly selecting their names from this database and contacting them via FRC email. 

### Data Analysis

#### Twitter Social Listening and Media Tracking

We conducted weekly quantitative and qualitative analyses of collected tweets. We evaluated top hashtags, expressing them as a percentage of weekly totals. Our qualitative analysis involved thematic coding of a random sample of all tweets (100 per week), from which we would identify the most frequently mentioned debates to raise in interviews. This random selection enabled us to pick up tweets, including conspiracy-related narratives, that might otherwise have been flagged or filtered out by an algorithm before they could trend.

An initial coding grid for tweets contained four major themes: risk perception, control, interpretations, and key actors and groups. To examine Twitter users’ perceptions of epidemic risk, we coded perceived viral origins, transmission and severity, and individual or social group susceptibility to the virus. For the control theme, we evaluated how Twitter users understood the efficacy, safety, and accessibility of preventive, diagnostic, and purportedly curative measures and devices (eg, masks, contact tracing, and distancing). The interpretations theme focused on narratives of viral origins and those profiteering from the pandemic. The key actors and groups theme categorized descriptions of specific actors or social groups.

Linked materials (eg, articles, other tweets, videos, and websites) were also evaluated for content. If the message alone was insufficiently clear, coders evaluated contextual tweets, as well as titles and descriptions of linked material. A short synthesis of contextual material was noted with the code to support thematic analysis. Two coders reached a coding agreement of Cohen κ=0.65 [58]. Divergent coding occurred when coders evaluated users’ perceptions of effectiveness of protective measures; in some cases, coders focused on the tweet’s content, and in others, on its broader context and linked content. Another disagreement was related to users’ attitudes toward protective measures (ie, those favoring or opposed to control measures and mandatory enforcement of masks or vaccines). Here, divergent coding resulted because control measures and mandatory enforcement (ie, masks, distancing, and limits on group sizes) were closely related. Coders discussed and, when necessary, modified code definitions to reflect consensus.

We consolidated biweekly social listening of pandemic discussions into short reports summarizing COVID-19 discussions. This analysis focuses primarily on the COVID-19 mask queries.

#### Interviews

All interviews were transcribed and integrated into NVivo software (2020 version; QSR International). The first author conducted the thematic coding of the interviews, building on the Twitter codebook by adding categories related to FRC activities, FRC actors’ motivation and engagement, and protective strategies. Team members wrote memos that formed the basis of our analysis by synthesizing coded content and detailing linkages across codes.

### Ethics

The protocol received ethical approval from the Institut Pasteur Institutional Review Board (IRB 2020-03) and was reviewed and approved by the FRC ethics committee. All study participants received an information notice and provided informed consent.

## Results

We collected and statistically analyzed 9,648,000 tweets, evaluated 1400 tweets qualitatively, and conducted 24 semistructured interviews with 8 FRC workers and 16 volunteers.

### Twitter Social Listening and Supplemental Media Tracking

#### Overview

Through Twitter-based social listening and supplemental media tracking, we followed discussions about COVID-19 control, risk perceptions, key actors and groups, and interpretations of purported origins and those profiteering from the pandemic. The supplemental media tracking guided our inquiries into newly emerging concerns on Twitter.

#### COVID-19 Risks

Between June and November 2020, many Twitter users discussed disease severity (n=127), most of whom (n=104) depicted COVID-19 as a dangerous disease, whereas a minority (n=23) described it as a mild disease affecting only the elderly; they compared it to seasonal influenza. The pandemic’s social consequences, particularly its social and economic costs and heightened exposures for marginalized social groups and frontline workers, were often emphasized (n=75). Uncertainties about the virus, specifically concerning transmission, mutations, and long-term consequences, also figured in discussions (n=59). A few users also shared diverging interpretations of the virus origins as being zoonotic, laboratory made, or 5G related (n=9).

#### Masks as Control Measures

Debates about the utility of masks, their scarcity, and changing policies regarding their use made masks the most discussed control measure for the study period in our social media–listening data.

Concerning the utility of masks, several users (n=14) echoed authorities’ and scientists’ calls to wear masks. Certain users (n=10) evoked COVID-19 susceptibility and severity as justification for mask wearing (n=10), whereas others (n=14) did so on the grounds that masks could prevent transmission, with a few contending that masks “would not be enough.”

Mask opponents, however, contested the legitimacy of political and scientific authorities who insisted on mask wearing (n=5), citing their own or others’ clinical experiences of mask use. Some also asserted that masks were ineffective: they “do not protect from COVID,” the virus “passes through” the mask, or masks “protect others but not yourself.” Two users emphasized the ineffectiveness of masks by observing that other countries successfully managed the pandemic without imposing masks or by insisting that handwashing was more important. Another two users claimed that COVID-19 would have no effect on them, one arguing that it was not a severe disease and the other claiming that the virus “did not exist.”

Debates over mask safety also emerged. Those considering masks to be safe (n=3) linked their claims to articles maintaining that masks did not reduce oxygen intake or asserted more generally that safety concerns had not surfaced over extensive mask use in the past. Mask opponents (n=4) countered claims of mask safety; some argued that they reduced oxygen intake—an especially serious concern during the summer, when the French state made them mandatory—whereas others contended that masks provoked skin conditions or were fabricated “in dirty places.”

Certain Twitter users criticized obligatory mask wearing as a ploy for political or economic gain. For some (n=5), mask regulations were a means of economic profiteering; the French government could “sell masks” or “collect fines,” retailers could become “rich” from mask proceeds, or “Chinese made the virus and sell the masks.” For others (n=11), masks were one tool in the arsenal of interests by politicians who sought to impose a “sanitary dictatorship,” to surveil citizens by embedding a “tracking device” in the masks or literally “muzzling” them.

Unequal access to masks also emerged as a subject of debate. Some attributed the mask shortage to poor governance (n=7) and others argued that masks should be distributed free of charge (n=11).

How social media users did or did not integrate masks into their daily lives was an additional debate. For some (n=8), masks were inconvenient in the workplace, on sunny days, and for makeup wearers. Others (n=4) found mask policies that recommended frequently changing or washing masks unrealistic; one offered tactics to evade mask use, and another claimed to wear masks to “avoid the fine.” Some users (n=10) argued about appropriate contexts for mask wearing, suggesting that they be worn only in closed spaces and by at-risk individuals and deploring constant reminders to wear masks. These arguments were countered by those who sought to make the best of mask wearing (n=3), maintaining that masks were a small price to pay for safety or offering tips for more comfortable mask wearing.

Some pro-mask users criticized inappropriate mask wearing (n=35), which included “no-maskers,” those wearing masks “under the nose” or “in the pocket,” and those who removed masks altogether to talk or smoke. They publicly denounced “no-maskers” (n=13), communicating descriptions of offenders to named authorities, recounting scenes of “no-maskers” driven out of public spaces, and deploring violence and resistance to calls for mask wearing.

Finally, state and regional changes in mask-wearing policies catalyzed many users to communicate new rules (n=44); some users interpreted these changes as governmental incoherence (n=8), whereas others (n=4) complained that traditional media outlets “talked too much” about masks (see Multimedia Appendix 2 for mask-related coding tree).

### In-depth Interviews

#### Interview Population

A total of 427 volunteers and workers were randomly invited from a contact database provided by the FRC to share their experiences anonymously and to provide recommendations for the organization. The response rate was 5.6% (24/427); 24 FRC workers and volunteers between the ages of 31 and 70 years participated in qualitative interviews (Table 1). Interviews did not collect data on the educational level of participants (see Multimedia Appendix 1 for the interview guide).

**Table 1:** Gender of interviewed French Red Cross workers and volunteers.

Gender	Participants (N=24), n (%)		
	Workers	Volunteers	Total
Women	5 (21)	10 (42)	15 (63)
Men	2 (8)	7 (29)	9 (38)
Total	7 (29)	17 (71)	24 (100)

#### Social and Traditional Media Provoked Anxiety, Uncertainty, and Disdain

All interviewees agreed that COVID-19 was dangerous for themselves, for the elderly, and for those with comorbidities. One-half contended that the persistence, volume, and contradictions in social and traditional media coverage cultivated deep uncertainty and/or anxiety. A few participants disdained claims of certainty expressed by social media users and commentators. Although some informants valued continuous media coverage encouraging public adherence to preventive measures or, in the case of social media, promoting FRC visibility or alternatives to traditional media coverage, most informants were critical. Social media networks, including Twitter and Facebook, came under particular criticism, reproached by FRC participants as uncontrolled sources and disseminators of rumors that undermined effective public health strategies. Social media users, they claimed, uncritically accepted rumors and, as a result, perceived themselves as an authority. Another critic, a retired industrial engineer and now FRC volunteer, lamented the following:

You know there is something that is hurting society badly nowadays, and that is social networks. It disintegrates society at the speed of lightning. So as soon as someone on a social network says, “The vaccine causes this, it causes that, there are risks, there are things,” it is seen, 10, 20, 30 thousand times and there you have it: it ignites.

Responses varied when we encouraged informants to identify how social and traditional media influenced their work offline during the pandemic. Some participants noted that internet-borne misinformation did not pose real-life problems for their daily field activities. Indeed, the retired engineer could not identify specific ways that social media had influenced FRC work, but nonetheless insisted that it did. He acknowledged, “I cannot link it to that...I could not tell you that there is an influence of social media or not...[although] I personally believe it.” Several other respondents, however, found that traditional and social media production fanned their disquiet. Media production was “anxiety-producing” (“anxiogène”), and several expressed disdain for the relentless criticism of experts, who seemed not to be concerned with the consequences of their remarks for listeners. One former hospital worker and volunteer observed the following:

I have never seen as many professors [as I have on TV] than during COVID. Every evening, there was a new one, saying, “We should not have done it this way, we should do it that way,” or “Not at all, we should have used x treatment,” [or] “It was not this, it was not that.” That was incredibly anxiety-producing for the average person...The epicenter was the unknown.

Other informants echoed this uncertainty and anxiety, bemoaning traditional media outlets’ desires to attract viewers and readers and to elicit their fuller engagement with disseminated information. One worker described media outlets as “horrendous,” always in “search for a buzz.” He opined, “Me, I find that horrendous...Because it puts people into a terrible state of mind, the term that summarizes everything: it’s anxiety-producing.”

Hence, several informants identified traditional and social media as exacerbating their uncertainty and anxiety, but also recognized that it remained difficult to pinpoint how social media and media productions influenced specific offline actions, either their own or those of other FRC workers.

#### “Tuning Out” as a Coping Strategy

One strategy reported by participants was to “tune out” traditional and social media, by “shutting down” their televisions or “logging off of Facebook.” For certain informants, turning off social and traditional media feeds resulted from an effort to reduce information-provoking anxiety and uncertainty. For others, turning off social media feeds stemmed from a desire to avoid excessive misleading information, or from a need to shut out unduly negative, repetitive claims. Hence, one volunteer claimed, “I cut off my Facebook and Twitter...I just stopped all that idiocy,” whereas another said of traditional media, “they [only] recounted the deaths, the hospitalizations, etc. I’d had enough...I didn’t want to listen anymore.” In such cases, workers and volunteers relied on the expertise of knowledgeable family members and the FRC to provide information and advice.

Turning off media feeds was not foolproof. One FRC worker who avoided social media feeds inadvertently found in her Facebook feed a news report suggesting a link between children with COVID-19 and Kawasaki syndrome, just as schools were about to reopen in France. At that point, she decided not to seek further information about the subject; she sent her children back to school and returned to volunteering with the FRC.

#### Traditional and Social Media: One of Multiple Influences on Decisions to Participate in Field Activities

The COVID-19 pandemic exacerbated needs among marginalized populations in the Paris region and imposed heightened demands on responding organizations, including the FRC, but multiple factors shaped individual workers’ and volunteers’ decisions to participate in FRC field activities that potentially brought them into contact with people suffering from the disease.

Social and traditional media coverage and commentary of ongoing FRC activities was one salient influence and, in some cases, galvanized our informants’ decisions to participate in field activities. Informants discussed television and social media depicting FRC support to transfer COVID-19 patients from overwhelmed hospitals to less busy ones, as well as the new *Croix-Rouge chez vous* (Red Cross at your home) platform, a call center–based service to deliver food and medicine to at-risk people confined to their homes and to provide psychological counseling. Workers and volunteers signaled that this media coverage heightened their desire to participate in field activities. Our informants underscored the pandemic as a singular, urgent, “historical” moment and their pride in contributing to FRC interventions. One volunteer noted, “It’s the first time for any volunteer who is alive today, at the Red Cross...at least in France we had never lived a crisis of such magnitude. We had all been trained since day 1 at the FRC for this kind of catastrophe.” A worker proclaimed,

We have all been very proud of what the Red Cross has done during this crisis. I think that whatever the domain, we have seen our colleagues in trains [patient transfers via high-speed railway, depicted on national television], in social outreach.

Nonetheless, decisions to return to FRC volunteering and work resulted from multiple factors. The influence of social and traditional media conjugated with perceptions of personal and familial health risks from exposure to COVID-19, family obligations concerning childcare and gendered expectations, income, employer pressures, and FRC regulations.

Our informants reported that FRC colleagues and family members relentlessly called and shared information over the phone and through messaging apps, such as WhatsApp, to influence their decisions and to remind them of familial obligations. Several participants faced active discouragement to participate in field activities by family members worrying about their health or that of other family members. One volunteer reported WhatsApp discussions of familial pressures among her fellow volunteers:

The most experienced [volunteers] asked themselves, “COVID, we don’t know what it is. I have a wife, I have kids, do I go into the field or not?” The fathers started a discussion...I think there were a couple of people under pressure from their wives, who were saying, “You shouldn’t go into the field.” So they would come to the crisis center [to volunteer] and then return home...to be there for snacks, baths, etc. They would “do the Red Cross” behind the scenes, and afterwards, in the evening, they’d be [at home] in “daddy mode.”

In addition to these familial obligations were gendered expectations. Several women volunteers and workers discontinued field activities because they had primary responsibility for childcare during school closures, although one man assumed childcare activities so that his wife could continue her field activities, even though this decision reduced family income.

Employer and related financial pressures were also important. A volunteer withdrew from FRC field activities at the request of her employer, who worried about workplace COVID-19 transmission. One worker received a request from the FRC not to work remotely, but instead to be paid for part-time unemployment because she had children to care for. Because the family could not cope financially with her partial unemployment, the worker and her husband found a family member to help care for the children.

Finally, certain volunteers did not decide at all: the FRC forbade volunteers aged 70 years and older, as well as volunteers and workers with existing comorbidities, to participate in any field-based activities. Although we heard reports of a few volunteers and workers circumventing these restrictions or participating through distant support, this measure made the decision for the volunteers and workers themselves.

#### Informational and Institutional Influences on Daily Protective Practices

For workers and volunteers engaging in FRC activities during the pandemic, how to carry out their work safely remained an important, daily consideration. Widely circulating, contradictory information about the virus and nonpharmaceutical prevention measures was just one factor shaping their everyday practices on the job or in the field. Other factors included state regulations, FRC institutional measures, communications and ethos, as well as coworker relationships and practices.

Several study participants noted that social media rumors and misinformation circulated among some FRC workers and volunteers, but they were divided about their effects on offline activities. Some contended that misinformation about mask effectiveness and its associated offline behaviors (eg, consequent refusals to wear masks) could pose problems for implementing certain activities. They also perceived misinformation attributing the origin or voluntary spread of the virus to a specific group as dangerous, for such claims contradicted the International Federation of the Red Cross principles of “humanity,” “unity,” and “universality.” Others considered this misinformation “ridiculous” and maintained that it would not affect volunteer or worker practice.

Multiple FRC institutional influences hindered the circulation of inaccurate information or diminished its effects on workers and volunteers and encouraged good protective practices to limit COVID-19 transmission (eg, mask use, physical distancing, and frequent handwashing). The FRC implemented an internal communication strategy, issuing bulletins and holding webinars to summarize information concerning protective equipment, other protective measures, and changing knowledge about SARS-CoV-2. It developed an internal “Frequently Asked Questions” page on its internal website to respond to worker and volunteer questions. Although FRC communications personnel recognized that workers and volunteers developed their own Facebook and WhatsApp groups, they built internal social networking tools and produced engaging and humorous social media content on the FRC action.

Several informants contended that the FRC institutional ethos, materialized in its uniform (Multimedia Appendix 3), compelled adherence to specific behaviors, including mask wearing. One male volunteer, when asked about how social media rumors about COVID-19 and protective measures might affect volunteers, reflected the following:

When you wear a uniform, I think that you execute the instructions that were given. There is a chain of command, and it is there to make sure that things will be respected. A uniform, I think that the moment we put it on, we put our personal opinions aside to focus on the mission.

Coworkers, notably those who had fallen ill with COVID-19 and returned to work, also influenced adherence to protective practices. An older volunteer noted the following:

It’s simple: everyone at the local unit knows that I was contaminated. I arrive in the morning with my mask on, so they see me arriving and say, “right, we have to wear the mask” [laughs]...but it does not come naturally; if I am not there, they don’t wear it...Because they did not have an experience of COVID, they don’t feel that the mask is of any use. The problem is that for a while, they were told that masks were useless, then they were told that they are useful but only in public transport, and, finally, they were told that they are useful everywhere. You see, it’s not easy for people to understand.

Yet not all our informants complied with FRC measures or coworker influences, reporting that normative measures could be negotiated in practice. Some, for instance, complained that FRC communication strategies contributed to the infodemic and exacerbated anxiety. One worker opined the following:

I think...that...[FRC management] should communicate about things that really happen, and not those that may not happen. All of those things that generate a huge amount of anguish and are never going to happen...In our service, for certain people, that generates a lot of anxiety...Instead, they should give themselves time to see what happens...to reflect and to put into place in collaboration with the teams, not to put the cart before the horse.

Echoing online and traditional media controversies, certain participants complained that some protective measures—floor distancing marks, one-way corridors, and disinfection routines around everyday objects—were not scientifically justifiable or realistic. Mask wearing preoccupied many informants, nearly half of whom expressed confusion about changing official discourses around masks. Actual mask wearing, they reported, varied considerably. One worker negotiated her mask-wearing practice through her daily interactions with coworkers. She noted, “I share the office with a colleague...I ask, ‘What do we do? Mask or no mask?’ I ask as if we needed to ensure that everyone present would be on the same page.” When asked if this was “a form of sanitary consent,” she responded, “Yes, that’s right.”

## Discussion

### Overview

**Figure 1:**
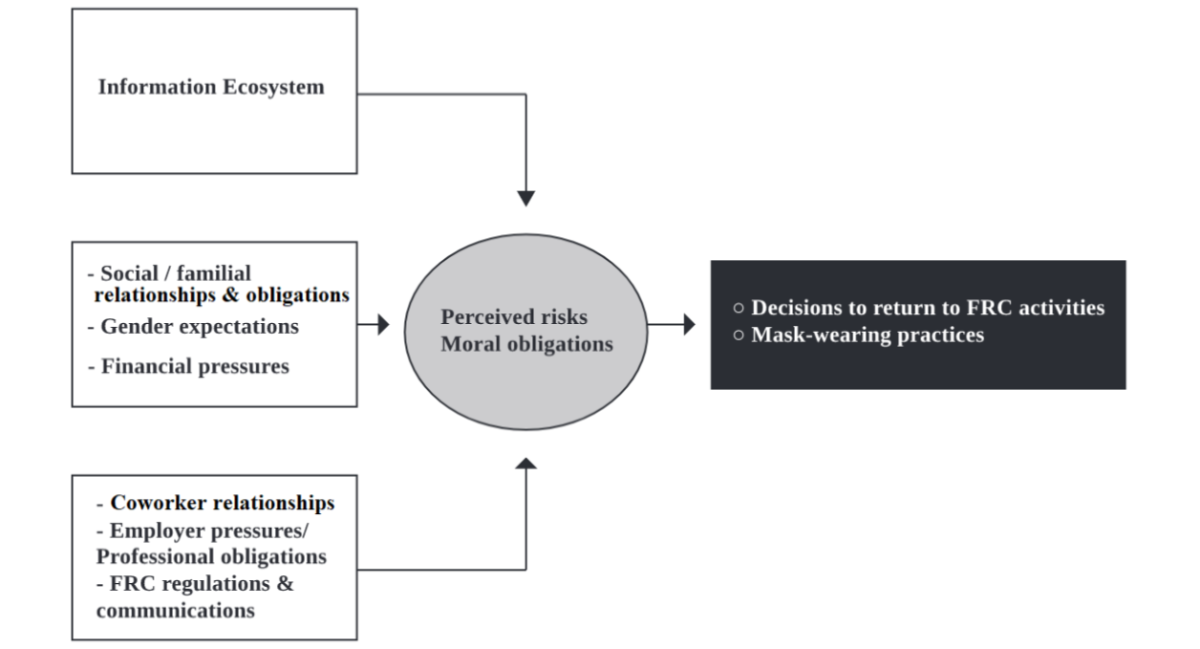
Factors shaping French Red Cross (FRC) volunteer and worker activity decisions and workplace masking practices.

Anthropological investigations of social media narratives are relatively few [8,59,60]; however, they fit into a much longer tradition of situating such narratives into social, political, economic, and historical relationships and understandings of rumors, risks, and practice [50,61-63]. This mixed methods study sought to determine whether the COVID-19 infodemic, particularly online and media debates about viral risks and protective measures, affected FRC workers’ and volunteers’ decisions to return to work and their protective practices in the Paris region. It analyzed two distinct data sets: (1) social media (Twitter) and selected traditional media and (2) qualitative interviews with volunteers and workers in one of France’s most important nongovernmental humanitarian assistance organizations, the FRC. Its contributions lie in an anthropological analysis of the influence of online debates around the virus, its risks, and protective masks on one group’s risk perceptions, decisions, and daily practices. We show that although online debates did affect FRC workers’ and volunteers’ emotions, decisions to return to work, and protective practices, other influences also played a role on their responses to the COVID-19 pandemic. Figure 1 summarizes the interactions between online COVID-19 debates and offline responses among FRC workers and volunteers in our study.

### Emotional Responses, Risk Perception, and Decision Making

We found that the COVID-19 infodemic incited anxiety, uncertainty, and, in some cases, disdain for expert opinions among interviewees. This response is coherent with other studies that found that social media can elicit emotional responses among users [28,29], including during the COVID-19 pandemic [35,38,39,43,64]. In some cases, FRC volunteers and workers protected themselves from the infodemic by shutting off social and traditional media or by relying on the FRC or on knowledgeable family members, colleagues, and friends. Although an adaptive response, tuning out and relying on social relationships can lead to less informed decision making and possibly worse health outcomes. New measures to reach such populations should be developed.

How the infodemic shaped FRC volunteers’ and workers’ risk perceptions and, ultimately, their decisions to return to field activities appears more complex. Although the information ecosystem provoked anxiety and uncertainty among our informants, other factors shaped their decisions: family, friends, colleagues and employers, financial concerns, and gendered expectations and norms influenced decisions to participate in FRC field activities. Risk perceptions and decisions to conduct field work did not simply entail epidemiological risks, but also social risks (ie, alienating family members encouraging one to return to the field or not), financial risks (ie, losing income), professional risks (ie, countering the wishes of one’s employer), and even a risk of losing one’s own sense of self-worth [63,65,66]. Our interviews suggested that the linkage between COVID-19 media debates and offline perceptions and decision making is far from straightforward. These decisions were contingent on the personal, familial, sociopolitical, and economic relationships in which volunteers and workers were embedded. Our anthropological lens thus contributes to prior studies of online influences on offline perceptions and behavior by accounting for these multiple factors in shaping decisions [8,54].

### Daily Protective Practices

Online social and traditional media debates around protection from COVID-19 comprised one factor among several that affected the daily protective practices of FRC volunteers and workers. Mask wearing could be inconsistent, which was explained by our informants as the consequence of a volatile, dynamic informational environment that, at times, discounted the effectiveness of masks. State regulations and FRC messaging and enforcement, in particular, did much to reinforce mask wearing among workers and volunteers, although our field evidence also suggests that masking could be a socially situated practice; an individual’s past history (eg, COVID-19 infection) and social relationships with coworkers or volunteers also played into mask-wearing practices.

### Anthropological Analyses of Infodemic Narratives

In contrast to many COVID-19 infodemic studies that conducted network or sentiment analyses [67-70], we employed an anthropological analysis of our evidence: we analyzed *all* claims, rather than triaging data as “true” or “false” and focusing solely on “false” information [19,21,71,72]. This approach has been useful in a pandemic, when biomedical and public health uncertainties about the virus have persisted and knowledge and policy have changed rapidly. Moreover, in evaluating epidemic narratives regardless of their truthfulness, we followed a useful anthropological tradition of exploring local understandings of risk, misfortune, sorcery, and the occult in their political, economic, and sociocultural contexts [45,73-77]. Examining the claims and circulation of epidemic narratives as neither true nor false helped us to better understand online narratives as well as their offline influences.

Evaluating only “false” narratives about masks would have neglected the changing narratives of masks in traditional and online media, the confusion that such changes precipitated, and their consequent erosion of public trust in health authorities and political leaders. Early in the pandemic, many authorities, including those in France, claimed that surgical or cloth masks would not prevent COVID-19 transmission and that lay publics would not use masks correctly. Moreover, multiple questions about transmission remained unresolved, including those about fomites, sexual contact, and aerosolized transmission [78-81]. In July 2020, French regulations around masks were implemented. Online debates questioning the effectiveness and necessity of masks reflect this changing knowledge, echoing Eysenbach’s observation that the early pandemic period must work with the “best evidence at the time,” not immutable claims to truth [6]. Claims that masks would usher in a “sanitary dictatorship” and surveil populations are not simply “false.” From an anthropological perspective, they yield rich insight into how certain French lay publics experienced current state public health measures for pandemic control as oppressive. Suspicions of state surveillance or ambitions of dictatorship did not spring forth suddenly in 2020 but have built on earlier political tensions. From 2017, the government has faced protests and massive strikes over retirement reforms, tax policy, police violence, budget cuts, and the climate crisis, among other concerns, as well as the rise of the *Gilets Jaunes* (“Yellow Vests”) as a new political opposition group [24,82,83]. Further examination of social and traditional media debates through an anthropological lens will be useful to open up specific online debates, to situate them in longer-term political, economic, and social tensions.

### Principal Results

This study found that social and traditional media were just one of many influences on FRC workers’ and volunteers’ decisions to work in the field and on their daily protective practices. FRC informants reported that social and traditional media provoked anxiety, uncertainty, and disdain for commentators’ claims to expertise. They also sought to “tune out” traditional and social media as a means of coping emotionally with persistent COVID-19 pandemic coverage.

### Limitations

This study has several limitations. First, French Twitter users and their beliefs, claims, or preoccupations are not representative of all populations in France or in the Paris region. For this reason, we undertook an iterative approach to media tracking, social listening, and interviews: selective media tracking influenced our social listening queries, and we used both as a proxy to identify major pandemic debates and a general typology or narratives about which we could ask our FRC informants during their interviews. Moreover, although captured tweets numbered in the millions, our qualitative analyses of a random sample of those tweets could only evaluate a small proportion of their online narratives. We were, thus, unable to address all online narratives through this analysis. Nevertheless, 1400 tweets are a substantial number for qualitative analysis, fitting into a range of similar thematic analyses of tweets [84-86].

Second, only a small proportion of FRC actors use social media, including Twitter, making it difficult to track how participants engaged with online information. We coupled our Twitter analysis, however, with selective media tracking to ensure that we had a proxy for major debates. This weakness is simultaneously an advantage, in that our informants experienced the informational environment as a complex, multi-sourced, contradictory onslaught of information, and not through the framework of a single social media platform.

Third, we were unable to conduct numerous interviews, although 24 is generally acceptable for publishable qualitative research. Our interviews do not reflect the perspectives of all FRC workers and volunteers in the Paris region. We initially hypothesized that our informants, because of their participation in a nongovernmental organization assisting people in humanitarian emergencies, were less likely to experience the influence of the infodemic, or perhaps less likely to admit to this influence. Recent literature, however, shows that frontline workers are highly likely to suffer from the infodemic and that US nurses were uncertain about or opposed to receiving COVID-19 vaccines [64,87]. Our qualitative approach helped to mitigate these limitations. The small sample size and the flexibility of our qualitative interviews allowed us to pursue lengthy conversations, cultivate nonjudgmental interactions, and build trust with informants during the interviews.

Anthropological interviews can yield rich data concerning how informants perceive risks, describe decision-making processes, and explain their protective behaviors, and they can situate these narratives and practices in a broad context of social, political, and economic relationships. They cannot, however, shed light on what people do in practice. Two lockdowns and the FRC’s heavy workload during the pandemic have hampered our efforts to undertake field observations of FRC activities. We supplemented our insights by meeting with FRC field actors and by the first author’s observations of FRC outreach actions.

### Conclusions

This study found that the social and traditional media narratives about COVID-19 and protective practices had an important emotional influence on interviewed FRC workers and volunteers. Excessive, rapidly circulating, and misleading information produced by the social and traditional media was only one of several factors, however, that affected FRC workers’ and volunteers’ decisions to contribute to field activities and to pursue daily protective practices, namely masking. Additional investigation of online narratives and expanded qualitative investigation, including observations of their offline influences among larger population samples, will be crucial to develop further insights. Moreover, measures to address users who have disengaged from online sources of health information and who rely on social relationships to obtain information are necessary. Tuning out can potentially lead to less informed decision making, leading to worse health outcomes.

## Appendix

Multimedia Appendix 1 Interview guide.

Multimedia Appendix 2 Mask codes and subcodes.

Multimedia Appendix 3 French Red Cross uniform.

## Abbreviations

FRC: French Red Cross
