# Supplementary material for: The Impact of the Online COVID-19 Infodemic on French Red Cross Actors’ Field Engagement and Protective Behaviors: Mixed Methods Study
Source: JMIR Infodemiology. 2021 Oct 6;1(1):e27472. doi: 10.2196/27472 (PMC8507423; doi:10.2196/27472)
Supplement: Multimedia Appendix 1 [file infodemiology_v1i1e27472_app1.docx]

**Multimedia Appendix 1**

**Interview guide**

**The interview is conducted as a semi-structured conversation between the researcher and informant. The interview guide is a highly flexible tool, so that the researcher can ask additional questions or provide additional prompts to the informant, depending on the responses provided.**

**Questions**

1. Please describe your background and how you came to work/volunteer for the French Red Cross?
2. Can you describe the nature of your work for the French Red Cross before the pandemic?

- Did you work with sick patients, with their families? Isolated/vulnerable people? the general public?
  - *Additional prompt*: What type of structure did you work in? What responsibilities did you have?

1. How has the pandemic has affected your activities?
2. **Have you received training or information from the French Red Cross to carry out your activities through an epidemic?**
   - ***Additional prompts:* What kind of information provided? With what frequency? How did you receive this information? Did you undergo mandatory training? Was it helpful?**
3. **Have you received resources to conduct this work during this epidemic?**  Do you think you are sufficiently equipped to carry out your mission?
   - *Additional prompts*: Did you obtain additional staff to conduct this work? Did you receive additional funds to do it? Did you receive personal protective equipment?
   - If so, why? Who provided these resources? Under what circumstances or conditions did this support take place?
   - If not, why not? What were the consequences ?
4. **Do you feel well prepared to care for patients/help vulnerable people during this epidemic?**

- ***Additional prompt:* Are you confident that you are able to implement the training you received?**
- ***Additional prompt:*** How did you evaluate your knowledge of coronavirus when you first began your mission with FRC during the pandemic? How has it changed over time?
- *Additional prompt*: Regarding your work on epidemic response, do you have any particular concerns or worries? (work or personal life)

1. **Have you taken on new responsibilities recently?**
   - ***Additional prompt:* Have you changed the way you work? How? Have you received any information about these changes, by what means?**
2. **How do you feel about carrying out your activities for FRC during the coronavirus epidemic? How does your family feel about it?**
   - ***Additional prompt:* Do you have any concerns? Which ones?**

**Do you feel motivated to continue? Why or why not?**

- - ***Additional prompt:***  What major challenges do you face in your FRC work? Are there solutions that could help you better address them?
  - ***Additional prompt:* How does your family feel about your involvement in the epidemic response?**
  - ***Additional prompt:* Have you changed your practices to protect yourself? (At home? work? Elsewhere?)**
  - ***Additional prompt:* What would you do if you caught COVID-19?**

1. **Are you in contact with other FRC actors (volunteers, salaried workers, supervisors) outside of work and volunteering hours?**

- **How do these exchanges take place? (WhatsApp, sms, social network, etc.)**
  - - **If so,** have you heard stories, informal information (or even rumors) about the disease, its origin, prevention, cures? (digging)
    - **If not, do you have another network with which you share your experiences?** Have you heard stories, informal information (or even rumors) about the disease, prevention, cures?

1. **During this study we are tracking social media (Twitter) and traditional media to understand more about debates concerning the virus and measures to control the epidemic.**

- **From where do you get your information about COVID-19? What sources do you consult?**
- **Are you active on any social media platforms? Why or why not?**
- **This week our tracking identified the following major debates (specify here):**
- **Have you noticed or participated in these debates online or among your acquaintances?**
- **Have you heard about these debates taking place within the Red Cross?**
  - **If yes, what did you hear? What different arguments did you learn about?**
  - **Did the FRC take a position on this debate?**
  - **Did you receive any guidance from the FRC about your activities or practices?**

1. **So far, do you think you/your team have responded well to this epidemic?**

- ***Additional prompt:* Can you report some activities that have been successful? Or others who have presented difficulties in their set up?**
- **Do you have any suggestions for improving the activities of the epidemic response?**

1. **In your experience, how have beneficiaries of FRC actions responded to this epidemic?**
   - ***Additional prompt:* Do they discuss COVID-19? Have you noticed any changes in behaviour during consultations/visits?**
   - *Additional prompt:* Do these beneficiaries ever recount to you any information, stories, or rumors? Can you give me some examples?
2. In terms of your work at FRC, what does "motivation" mean to you? Why do you work for FRC?
