# Supplementary figures and images for: The Impact of the Online COVID-19 Infodemic on French Red Cross Actors’ Field Engagement and Protective Behaviors: Mixed Methods Study
Source: JMIR Infodemiology. 2021 Oct 6;1(1):e27472. doi: 10.2196/27472 (PMC8507423; doi:10.2196/27472)

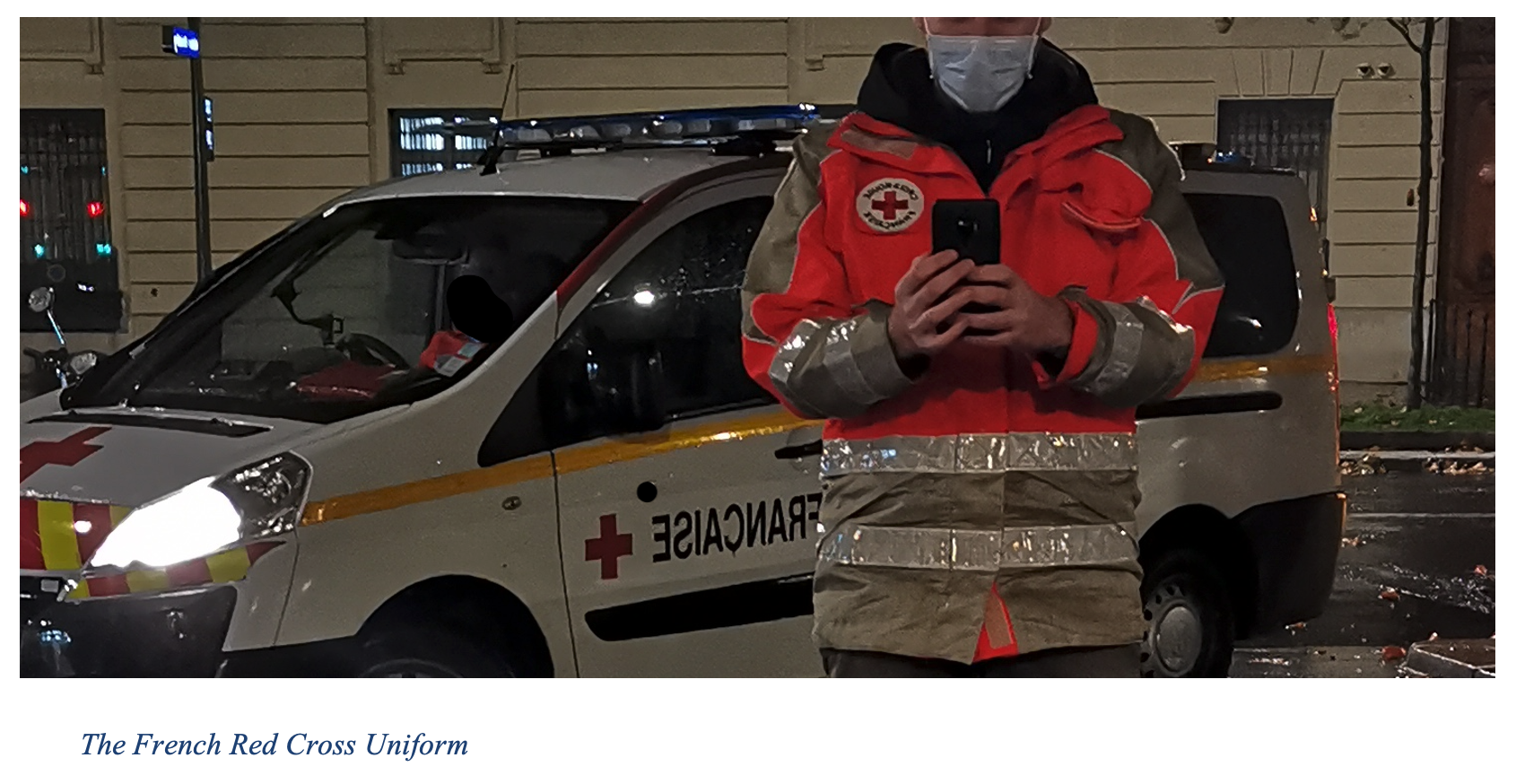

Supplement: Multimedia Appendix 3 [file infodemiology_v1i1e27472_app3.png]
